# Mourning for Silence: Bereavement and Tinnitus—A Perspective

**DOI:** 10.3390/jcm14072218

**Published:** 2025-03-25

**Authors:** Dirk De Ridder, Berthold Langguth, Winfried Schlee

**Affiliations:** 1 Section of Neurosurgery, Department of Surgical Sciences, University of Otago, Dunedin 9016, New Zealand; 2 Department of Psychiatry and Psychotherapy, University of Regensburg, 93053 Regensburg, Germany; berthold.langguth@medbo.de; 3 Institute for Information and Process Management, Eastern Switzerland University of Applied Sciences, 9000 St. Gallen, Switzerland; winfried.schlee@ost.ch

**Keywords:** tinnitus, bereavement, trajectory

## Abstract

Tinnitus is defined as the conscious awareness of a tonal or composite noise for which there is no identifiable corresponding external acoustic source, which becomes tinnitus disorder when the phantom sound is associated with suffering and/or disability. There is only limited knowledge about the time course of tinnitus disorder. Bereavement science has identified four different trajectories: resilience, recovery, chronic, and delayed. The question arises whether these four trajectories exist in tinnitus as well if one considers tinnitus as the loss of silence (at will). To verify whether these four trajectories exist, short-term tinnitus progression was analyzed retrospectively using an Ecological Momentary Assessment (EMA) approach, extracting the data from patients who started using the TrackYourTinnitus (TYT) app (version 1, Ulm University, 2013) from the start of their tinnitus perception. Four patients were identified retrospectively via the TYT app with acute tinnitus, and the bereavement trajectories were reconstructed based on EMA. In conclusion, this perspective suggests that the four known bereavement trajectories may exist in tinnitus, and prospective evaluations of larger samples are warranted to confirm or disprove this analogy between bereavement and tinnitus, in which tinnitus is conceived as the loss of (controllable) silence.

## 1. Introduction

Tinnitus has been defined as the conscious awareness of a tonal or composite noise for which there is no identifiable corresponding external acoustic source, which becomes tinnitus disorder when associated with emotional distress, cognitive dysfunction, and/or autonomic arousal, leading to behavioral changes and functional disability [1]. In other words, “tinnitus” describes the auditory or sensory component, whereas “tinnitus disorder” reflects the auditory component with associated suffering [1]. These definitions are based on an analogy of pain [2,3] and adapted from definitions already in use in the DSM-V and ICD11 for pain [1]. Both tinnitus and pain have similar neurophilosophical (Darwinian and Bayesian), anatomical (lateral, medial and descending pathways), pathophysiological (thalamocortical dysrhythmia), clinical (entirely subjective, wind-up, and hypersensitivity), and treatment (psychology, medication, neuromodulation, and surgery) characteristics [4,5,6,7,8], permitting the definitions’ alignment. As in tinnitus, in pain, there is a sensory component and a suffering component [8,9], leading to the classification of Somatic Symptom Disorder in the DSM-5, previously known as pain disorder. However, in contrast to pain, in which the ICD 11 classification recognizes seven subgroups [10,11], no subgroups have yet been defined despite multiple statistical attempts. This may mean that either tinnitus is not as heterogeneous as once thought from a ‘pathophysiological’ point of view, or some crucial aspects are missing. In tinnitus, large-scale ‘clinical’ heterogeneity has resulted in problems of standardizing diagnosis, management, and treatment. Four main dimensions have been described for tinnitus heterogeneity: 1. perception; 2. risk factors/comorbidities; 3. distress/suffering; and 4. treatment responses [12]. However, this assumes that all tinnitus is inherently static in nature, which it is not. Tinnitus can change over time, which means a fifth dimension needs to be added to clinical heterogeneity. Looking at the natural evolution of tinnitus, it has been shown that while in 80% of patients it is relatively static, in 20% of patients, tinnitus can disappear within 4 years, and in those 80% in whom it persists, in 10%, it can improve, and in 10%, it can worsen [13]. This suggests that at least four different trajectories exist: recovery, delayed worsening, and stability (resilient or chronic), further adding to the heterogeneity of tinnitus. The prevalence of tinnitus in the general population is estimated at 14.4% [14]. Whereas about 80% of people who have tinnitus can cope with it and can lead normal lives, for about 20% of people, tinnitus is associated with suffering [14,15].

For individuals exposed to work-related noise, prevalence is estimated at 23.3% [16]. In the military, 6–7% of soldiers develop tinnitus after deployment [17]. Depending upon the circumstances, traumatic events may contribute to the onset of tinnitus [18,19,20,21]. In veterans, the prevalence of severe and very severe tinnitus (= tinnitus disorder) is significantly higher, and this is related to PTSD. Indeed, veterans with tinnitus, but without PTSD, typically report predominantly mild tinnitus, whereas veterans with PTSD typically report moderate, severe, or very severe tinnitus, and this is associated with increased anxiety and depression [22]. Among PTSD-affected soldiers, the prevalence of tinnitus is over 50% [23]. PTSD is considered a psychological injury, and while tinnitus is a symptom, its onset may be connected in memory to the injury, thereby evincing the capacity to exacerbate the trauma’s effects [20].

Tinnitus and depression are comorbid, as evidenced in veterans [22], but also in the general public [24]. Whereas acute tinnitus is comorbid with anxiety and insomnia, chronic tinnitus is more often associated with depression [25].

This suggests that tinnitus perception may change over time [26,27], associated with changes in the neural signature of tinnitus in the brain, which also changes over time [28]. Most studies that aimed to investigate the time course of tinnitus assumed a common progression. However, based on bereavement science, it can be hypothesized that patients may follow any of four trajectories describing grief due to bereavement [29]. This means that tinnitus can be conceptualized to lead to the feeling of bereavement or loss of a bygone era in which there was no phantom sound, an era characterized by silence that could be controlled at will. Thus, tinnitus may lead to the feeling of mourning for the loss of silence, and hence researchers in the tinnitus field may learn something from bereavement science.

Bereavement science demonstrates that there are four trajectories to trauma and grief: 1. resilience, 2. recovery, 3. chronic dysfunction, and 4. delayed trauma [29]. These trajectories are similar for prolonged grief disorder, PTSD, and depression [30], i.e., when a close family member dies, people are either resilient to prolonged grief, PTSD, and depression, or recover from all four or not [30]. Considering that PTSD and depression are comorbid to tinnitus in ± 25% of people [24], it can be hypothesized that if tinnitus is considered as a trauma or the bereavement of controllable silence, these four trajectories could also exist in tinnitus and help to distinguish between tinnitus and tinnitus disorder.

Resilient people continue to thrive irrespective of trauma or loss. Their normal functioning is not or only mildly disrupted. In recovery, people’s daily functioning is initially moderately or severely disrupted, but over 1 to 2 years there is the gradual normalization of functioning. Chronic dysfunction is characterized by the severe disruption of normal functioning from the beginning, which does not improve over time. Delayed trauma leads initially to mild or moderate disruption of normal functioning, which after a delay worsens progressively to severe interference [29].

The question is whether these trajectories also exist in tinnitus. But irrespective of this, researchers in the tinnitus field can learn from bereavement science that tinnitus heterogeneity may be linked to these different trajectories, which means the tinnitus field may benefit from a similar stratification.

## 2. Methods and Materials

To verify whether these 4 trajectories may possibly exist, as posited in this perspective, we explored tinnitus progression retrospectively using Ecological Momentary Assessment (EMA), a commonly used research method that aims to assess phenomena with a focus on ecological validity and to help both the user and the researcher observe these phenomena over time. One phenomenon that benefits from this capability is chronic tinnitus. TrackYourTinnitus (TYT) (version 1, Ulm University, 2013) is an EMA-based mobile crowdsensing platform designed to provide more insights into tinnitus by repeatedly assessing various dimensions of tinnitus, including perception (i.e., perceived presence).

Using the TrackYourTinnitus database, 4 patients were retrospectively retrieved who started charting tinnitus loudness and tinnitus annoyance almost from day 1 of developing tinnitus.

The TrackYourTinnitus study was conducted according to the guidelines of the Declaration of Helsinki and approved by the Institutional Ethics Committee of the University of Regensburg (protocol number: 15-101-0204) dated 26 August 2015.

## 3. Results

**Figure 1:**
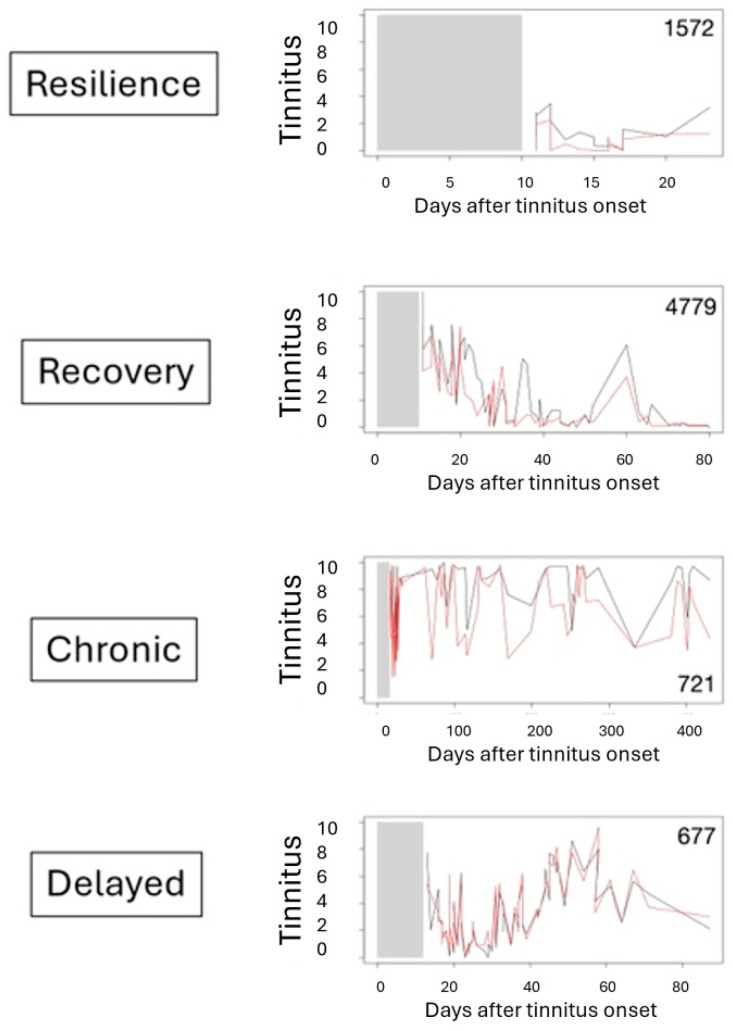
Trajectories of patients with tinnitus. Red line = tinnitus loudness; black line = tinnitus distress.

Of the four patients who charted tinnitus from the acute setting, one was defined as being resilient (1572), one as following a quick recovery trajectory (4479), one as following a chronic trajectory (721), and one as having a delayed trajectory (677) (see Figure 1 and Table 1). Since people who use the TYT app are not required to provide all the requested data, the demographic and clinical data are only partial (see Table 1). Their ages were between 42 and 62 years old, three of four people were male, and the miniTQ score varied from five to twenty-three (from no distress to most severe distress). The resilient trajectory is associated with the absence of distress. Recovery occurred in the patient who initially had the most severe distress, which also is seen in the delayed trajectory. The chronic trajectory was observed in the patient with severe distress, but not the most severe distress and also not moderate nor the absence of distress (obviously).

## 4. Discussion

Tinnitus is well known as a heterogenous disorder with multiple hypothesized subtypes [33]. Many researchers and clinicians have tried to classify these subtypes according to their clinical profiles, etiologies, and response to treatment, with little success [33]. The occurrence of overlapping tinnitus subtypes suggests that the disorder exists along a continuum of severity, with no clear distinct boundaries [33]. From a mechanistic point of view, the unified, personally unique tinnitus percept has been described as an emergent property of partially overlapping, dynamically changing, interacting brain networks, each representing a different clinical aspect of the unified, individualized tinnitus percept [34], suggesting that the interaction of these separate networks determines the phenomenology of tinnitus, ultimately leading to a dimensional spectrum, rather than categorical subtypes [33]. The question is whether there exists one common trajectory all patients go through, or whether multiple different trajectories exist?

**Figure 2:**
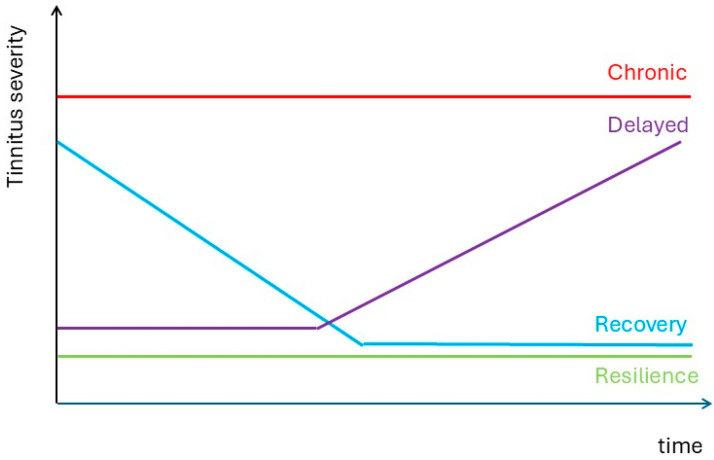
Schematized trajectories of tinnitus evolution based on bereavement science.

Based on this small exemplary data sample of tinnitus patients who filled out an EMA form using the TrackYourTinnitus app from the early stages of acute tinnitus shows that the four trajectories known in bereavement science may also be present in patients with tinnitus (Figure 2, Table 2). Thus, conceptually, in tinnitus, we may see the 4 suggested trajectories.

### 4.1. Resilience (±70–80%) [30]

Resilience is defined as the capacity of a dynamic system to adapt successfully to disturbances that threaten system function, viability, or development [35]. Applied to neuroscience, it is defined as the ability to cognitively or emotionally cope with stress, trauma, or adversity without long-term negative consequences. Psychological resilience is present in 80% of people with bereavement [30] and PTSD [36]. This is similar to the 85% of patients who have tinnitus, but do not suffer from it [14,15]. Resilience has a protective effect on tinnitus handicap, as indexed by the THI, and neuroticism has a negative effect [37]. Thus, people with high psychological resilience will tolerate the presence of tinnitus better, which is in keeping with bereavement science. Resilience may not have a direct or indirect influence on tinnitus loudness [38]. However, resilience influences annoyance directly, as does neuroticism on tinnitus annoyance and severity [38,39]. The protective effect of high resilience is associated with better emotional health and reduced depression, anxiety, and somatic symptom severity, which, in turn, are associated with a less distressing tinnitus [40]. Based on the natural history of tinnitus, it can be estimated that resilience and recovery is indeed present in 80% of people with tinnitus [13]. This is similar to what is known about trauma. Meta-analyses have shown that around 80% of trauma-exposed people are resilient to or recover from trauma, whether it is related to major traumatic events [36], floods [41], war zones [42], or pandemics [43]. This is also similar to prolonged grief disorder [44], although the incidence of the latter varies between 10% [45] and 40% [46], which may be due to the very low certainty level of the evidence for the pooled prevalence of Prolonged Grief Disorder (PGD) and its symptoms [47].

### 4.2. Recovery (±10–20%) [30]: Normal Transient Suffering

Suffering is normal in bereavement, and when resilience does not prevent the impact of suffering on normal functioning, it can lead to transient disability. However, this can be followed by recovery, taking 1–2 years [29,48,49]. In tinnitus, in about 20% of patients the THI score improves by twenty points, and about 50% by seven points (=Minimal Clinically Important Difference, MCID) over a period of 1–6 years. As the patients did seek treatment, so it cannot be concluded whether this is solely based on spontaneous recovery [27]. However, natural evolution demonstrates that after 4 years, 20% of people with tinnitus no longer have it, and in the 80% of patients who still have tinnitus, in 10% there is an improvement [13]. Furthermore, complete recovery from tinnitus is possible even in chronic cases [50].

### 4.3. Chronic (±5%) [30]: Pathological Persistent Suffering = Tinnitus Disorder

Chronic suffering as expressed as prolonged grief and PTSD symptoms, and depression is present in 5% of bereaved patients [30]. This is similar in magnitude to the global prevalence of tinnitus disorder (2.3%) [14]. The risk factors for these bereaved patients who are chronically suffering are neuroticism [51,52] and insomnia [53], whereas social support is protective [51]. Comorbid anxiety, depression, and PTSD result in more grief [54]. In tinnitus, the risk factors for bothersome tinnitus are also neuroticism and insomnia [13,55] and also include hearing difficulties, ototoxic medication, and work noise exposure [13,55]. It is important that tinnitus loudness is not the principal determinant of whether people suffer chronically, but rather their personality characteristics, sleep behaviors, and hearing difficulties.

### 4.4. Delayed (±5%) [30] Tinnitus Disorder

The delayed worsening of bereavement is rare, and it is unclear whether this really exists [56]. In tinnitus, this is also unknown. Delayed tinnitus has been hypothesized to exist in those with noise trauma [57,58] according to evidence based on rat studies [59]. Mechanistically, this may be related to an inflammatory response that develops progressively in the auditory system associated with noise trauma [60]. From a theoretical perspective, a second mechanism could involve patients who could initially cope with the tinnitus but are exposed to emotional stress for other reasons. When the extra stressors occur, they may experience worsening of pre-existing tinnitus and tinnitus severity [61]. Usually, this will only result in transient worsening or fluctuation, but if the stressors are severe or repetitive, this could lead to persistent worsening. A summary of these four categories can be found in Table 2.

In all these trajectories, temporarily fluctuations may occur, when people suffer more and/or perceive the phantom sound louder, but this worsening is only transient and commonly associated with any extra non-specific stressors. Indeed, ‘suffering’ is processed in the brain in the medial pathway, which is non-specific, i.e., it is the same for tinnitus, pain, or any other stimulus, and overlaps with the stress network [8,9,62,63]. Thus, any stress may worsen tinnitus-associated suffering. Furthermore, stress changes the sensory gain via increased alpha2 noradrenergic receptor activation [64], which could make patients perceive tinnitus louder. Stress that transiently worsens tinnitus can be social, e.g., work- or family-related; associated with physical stress, such as surgeries or infections; or psychological. Stress may exacerbate both tinnitus loudness and tinnitus-associated suffering transiently via its effect on the medial network, which colocalizes with the salience network (insula, ACC) in cognitive neuroscience [9,65]. Normally after the stressor is removed, the patients will recover to their previous state. However, more chronic stress may create longer-lasting worsening by its immune dysfunction-inducing effect [66], and its interference with the triple network may reflect a double hit as well [67], which has been posited to be crucially involved in tinnitus processing [63]. It is indeed known that stress in general can worsen the tinnitus percept [61,68,69].

Stress increases daily energy expenditure by 30%, as does immune activation, and when stress results in insomnia, this may add another 30% extra energy expenditure [70]. This will lead to fatigue and mental exhaustion. Consequently, it is unsurprising that fatigue is associated with higher tinnitus-related distress [71].

Is there a theoretical framework for this analogy?

There exist multiple brain-based theoretical models of tinnitus and tinnitus disorder. No coherent encompassing model exists yet that integrates different tinnitus models, such as the gain model [72], the neuroinflammation model [73], the multiple network model [74], the imbalance model [8], the Bayesian brain models [75,76], and the triple network model [63]. As seen from the variations in the Bayesian brain model, it can be proposed that when auditory deafferentation occurs, with or without hearing loss [77], this will result in auditory uncertainty about what occurs in the changing environment [76]. When this auditory deprivation is not deemed salient, no tinnitus will be generated [78], yet when the brain does consider the lack of auditory input as salient, it follows a better-safe-than-sorry approach and fills in the missing auditory information by generating the sound itself, thereby reducing auditory uncertainty [4]. As such, tinnitus can be seen as an uncertainty disorder [79], which can generate stress and suffering in people who are genetically or epigenetically prone. Reducing auditory uncertainty via a filling-in mechanism may explain the development of tinnitus [76]. We now propose that the based on this theoretical foundation, tinnitus may generate a feeling of bereavement of the ‘loss of silence’ at will. Everybody who develops tinnitus may initially perceive bereavement as the feeling of loss of silence at will, but depending on individual coping mechanisms this may result in different trajectories that are akin to what has been theorized in the bereavement literature [56]. Indeed, when hearing loss is associated with neuroticism, sleep deprivation, work noise exposure, or ototoxic drugs, this increases the risk for developing bothersome tinnitus [13], which could evolve into a disorder, i.e., tinnitus with suffering, when bother turns into anxiety and depression. As such, tinnitus is the outcome of a resilient or recovery trajectory, and tinnitus disorder is the consequence of a chronic or delayed trajectory. In essence, the perceived distress or intrusiveness associated with tinnitus may theoretically reflect maladaptive coping with auditory uncertainty. In other words, not accepting that there may never be silence (at will) anymore can lead to tinnitus disorder, which is similar to not accepting that the loss of a loved one is forever, leading to complicated grief or prolonged grief disorder. From this point of view, the acceptance of bereavement, i.e., the ‘loss of silence’, is inversely proportional to perceived intrusiveness, distress, or suffering.

What drives certain patients to follow one trajectory, and others a different one?

Resilience is the default response to the development of tinnitus, as it occurs in 80% of people with tinnitus. Resilience is based on genetic and epigenetic factors that involve the nervous system, the immune system, and the endocrine system [80], all involved in integrated and concerted responses to internal and external stressors [66]. Indeed, for PTSD, it has been shown that if someone has the risk genes for PTSD, a single incidence of exposure to trauma is sufficient to trigger the development of PTSD by activating PTSD risk gene expression [81,82]. However, also some people without the risk genes develop PTSD after trauma. This has been linked to epigenetic tagging, e.g., via the methylation of the same genes, turning normal genes into functional risk genes [81]. Epigenetic tagging can for example occur as a consequence of an extreme stressor in early childhood, such as any form of severe childhood adversity. But epigenetic modifications can result from other causes, such as toxins, medication, diet, psychological or physical stress, and trauma [9]. In other words, with the risk genes for poor resilience, a person may end up on a trajectory that differs from the resilience trajectory, such as the chronic distress trajectory or the delayed trajectory. But a person without those risk genes may still end up on the chronic or risk trajectory if there have been epigenetic modifications to the resilience genes due to early childhood adversity, psychological or physical trauma, intoxications, poor diet, medication, etc., resulting in a double hit [81,82]. In other words, people without risk genes require two traumas for PTSD to develop. An analogy of tinnitus would be that hearing loss only triggers tinnitus if certain risk genes are present, or if two hits occur, in which the first hit triggers the epigenetic tagging of the tinnitus risk genes, and the second hit subsequently triggers tinnitus. This has been suggested in people with polymorphism for the COMT gene. COMT Val158Met polymorphism can increase susceptibility to the clinical manifestation of tinnitus in those people with hearing loss [83]. However, this does not result in suffering. For this, another polymorphism may be essential. BDNF Val66Met polymorphism can result in suffering with the associated worsening of tinnitus loudness perception [84]. This may need to be seen from a broad perspective. Some genetic polymorphisms are associated with clinical features. The genes associated with tinnitus and hearing loss overlap partially, but some are different [85]. Also, personality characteristics such as neuroticism are genetically determined [86,87], as is long and short sleep duration [88], and insomnia [89]. All these factors increase the risk for people with tinnitus to follow the chronic suffering trajectory. Neuroticism is indeed a risk factor for chronic grief/distress [51] and tinnitus distress [90,91]. Some polymorphisms associated with neuroticism overlap with the genes involved in resilience, suggesting that indeed genetic and epigenetic influences partially determine which trajectory a person will follow once tinnitus occurs [92]. Considering that neuroticism genes also overlap with anxiety and depression risk genes [87], this may explain why people with genetic risk genes for neuroticism may have an increased risk of developing tinnitus disorder (= the trajectory of chronic distressed tinnitus) [13,37,39]. Insomnia is also a risk factor for developing the chronic trajectory. Insomnia can lead to immune dysfunction [70,93,94], triggering a long-term neuroinflammatory pathology which is known to be associated with most chronic neurological and psychiatric disorders, including anxiety [95], depression [95], tinnitus [73,96,97], and PTSD [98,99].

**Figure 3:**
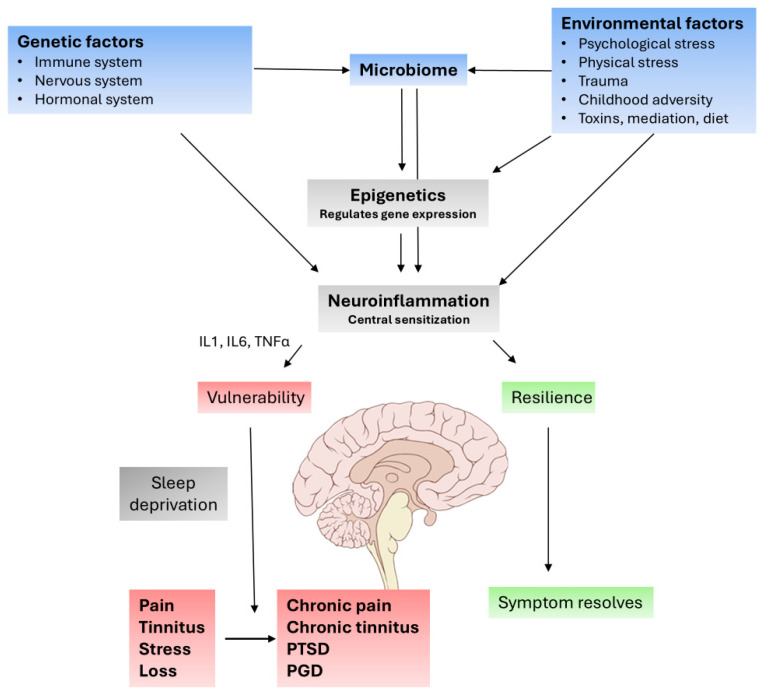
Similar pathophysiological mechanisms underpin chronic pain, chronic tinnitus, PTSD and PGD, in which genetic vulnerability and environment-induced epigenetic changes introduce proinflammatory state that turns the acute symptom into a chronic persistent state.

In summary, tinnitus, pain, PTSD, and PGD may share a common pathophysiological mechanism, in which genetic and environmental (epigenetic) factors determine whether low-grade neuroinflammation turns an acute symptom, such as pain, tinnitus, stress, or loss, in a chronic state, resulting in chronic pain, chronic tinnitus, PTSD, and PGD (= complicated grief). This may be mediated or worsened via the sleep problems common to all these pathologies. The analogy involving these four disorders is summarized in Figure 3, based on the proposed very similar pathophysiological mechanisms involved in chronic pain [9], chronic tinnitus, PTSD [100], and bereavement [101].

Are these trajectories clinically meaningful?

The four different trajectories may require different management and treatment approaches, suggesting that incorporating these trajectories in studies may be highly relevant to help combat the heterogeneity of populations included in studies, as well for individual patients seen in the clinic. These trajectories do suggest that it may even be useful to create different tinnitus management programs and treatment centers for the different trajectories. Whereas those with resilience do not require treatment, patients on the recovery trajectory may benefit from simple short-term audiological/ENT or psychological management to facilitate the natural recovery process. On the other hand, those on the chronic and delayed trajectories may require long-term multidisciplinary help involving multidisciplinary tinnitus centers with neurology/neurosurgery/psychiatry/psychology and ENT/audiology collaboration. People on the chronic trajectory may require multimodal approaches, such as a combination of CBT, medication, and/or neuromodulation for comorbid suffering involving sleep problems, anxiety, and depression. This may need to be maintained if, for example, the chronic trajectory is due to factors that are not sufficiently addressed or cannot be addressed such as neuroticism, ototoxic medication, a loud workplace, and progressive hearing loss. The delayed group is especially intriguing as it can be envisioned that timely CBT or multimodal treatment could prevent the transition from tinnitus to tinnitus disorder.

Whereas resilience is related to well-characterized genes [80,92], complicated grief (chronic and delayed trajectories) after the loss of a spouse is linked to different gene variants [101], especially related to inflammation, e.g., via IL6-174 SNP [102] and the gene variants that downregulate interferon 1 immune responses [103]. This has resulted in a model that suggests that bereavement trajectories differ based on a genetically and epigenetically induced proinflammatory state [101], analogous to what has been proposed for tinnitus [73,97].

Different genes have been linked to the development of tinnitus and tinnitus disorder, strongly suggesting that the clinical distinction between tinnitus and tinnitus disorder is reflected in a different genetic structure [104,105]. The rare genetic variants associated with tinnitus disorder include those related to neural activity (e.g., *ANK2*, *NAV2*, and *TMEM132D*), inflammation (e.g., *TSC2*), metabolism (e.g., *AKAP9*), and calcium channel function (*CACNA1E*) [104,105]. As tinnitus disorder is typical for chronic and delayed trajectories, it may be of relevance to look at inflammation-related genes, as neuroinflammation is involved in tinnitus, complicated grief, anxiety [96], and depression [95]. These variants differ from the common variants found in tinnitus GWAS, highlighting the distinct genetic underpinnings for tinnitus disorder compared to those of tinnitus alone. Furthermore, some genes involved in tinnitus disorder such as *TMEM132* have also been associated with anxiety [106,107], explaining that these gene variants are relevant for tinnitus disorder. In summary, it can be proposed that some gene variants make people vulnerable to develop tinnitus in the setting of auditory uncertainty related to deafferentation, and having some other extra, but different gene variants may determine whether tinnitus results in associated suffering (tinnitus disorder) due to neuroinflammatory processes.

As a consequence, involving geneticists and pharmacologists, especially for treating people who follow the chronic or delayed trajectory should be arranged. For example, genetic studies show that six druggable genes (TLR4, MMRN1, BRAF, ACVR1B, NOS2, and GPX1) are already being explored for drug development targeting neuroticism [86], which may be used in the early treatment of people at risk of following the chronic trajectory. For example, TLR4 is targeted by cyclobenzaprine [86], which is currently used to treat tinnitus [108], NOS2 can be targeted by dexamethasone and gingko biloba [73], which are also already used to treat tinnitus [109]. NOS2 can also be targeted by arginine, miconazole, and minocycline [86], which could theoretically be repurposed for the treatment of tinnitus. And GPX1 is targeted by cannabidiol, but also glutathione and selenium [86], which are readily available. Thus, understanding the genetic and non-genetic risk factors for these trajectories could potentially lead to more personalized management and treatment approaches in people suffering from tinnitus.

A weakness of this perspective is that it could be a self-fulfilling prophecy by specifically searching for the expected trajectories. While this could be a correct assumption, this is the case with all theoretical models. Another weakness is that tinnitus tracking was of a short duration. Consequently, what is fitted to the chronic trajectory in this representative case may still be the recovery trajectory in the long term.

How do we move forward?

Ideally, a large prospective long-term follow up dataset needs to be developed that tracks many patients with tinnitus from day 1 for at least 1 year longitudinally for tinnitus loudness, tinnitus-associated distress/suffering, and disability, in which, subsequently, data-driven cluster analysis is performed to extract the statistically independent trajectories.

Once this stratification is performed, genetic and epigenetic studies may add to the clinical data by helping to develop personalized treatment approaches for patients. This may require multidisciplinary tinnitus centers, analogous to what already exists for pain.

## 5. Conclusions

If tinnitus is addressed from the point of view that tinnitus represents the loss of silence (at will), and thus bereavement, it can be theorized that the canonical four trajectories of bereavement may exist in tinnitus. And even though this study is based on little data, the four trajectories that exist in bereavement are compatible with the tinnitus trajectories. It is evident that all the trajectories may fluctuate in the long term, with ups and downs depending on the non-tinnitus-related extra stressors transiently worsening tinnitus loudness and annoyance. Recognizing these trajectories may result in the more personalized management and treatment of tinnitus. Further prospective studies looking at larger samples may prove or disprove this theoretical perspective.

## Figures and Tables

**Table 1:** Demographic and clinical characteristics of the four patients.

User ID	1572 Resilient	4779 Recovery	721 Chronic	677 Delayed
age	55	62	62	42
sex	male	male	female	male
miniTQ	5 (no distress)	23 (most severe)	15 (severe)	20 (most severe)
tinnitus side	right ear	right ear	inside head	both ears, worse in left ear
description of tone	high-frequency whistle	high-frequency tone	crickets	tone
vertigo	NA	no	NA	NA
hearing problem	NA	no	NA	NA
hearing aids	NA	no	NA	NA
headache	NA	no	NA	NA
psychiatric treatment	NA	no	NA	NA
medication	NA	no	NA	NA

Questionnaires used: TSCHQ = Tinnitus Sample Case History Questionnaire [31] and miniTQ = mini Tinnitus Questionnaire) [32], NA = data Not Available.

**Table 2:** Bereavement trajectories applied to tinnitus.

Trajectory	Bereavement	Tinnitus (Loss of Silence)
**Resilience**:	Minimal disruption in daily functioning	Tinnitus causes minimal disruption in daily functioning
	Low levels of distress or grief symptoms	Acceptance of tinnitus as a non-threatening aspect of their sensory experience
	Adaptive coping skills	Individuals adapt very quickly to the presence of tinnitus using adaptive coping skills
**Recovery:**	Intense emotional pain early on	Initially high levels of annoyance, anxiety, or emotional pain
	Gradual improvement in mood and functioning	Gradual reduction in distress as habituation occurs or therapeutic interventions are successful to reduce tinnitus
	Effective use of support systems and coping mechanisms	Return to a baseline sense of control over life.
**Chronic grief:**	Long-lasting emotional pain and difficulty adjusting to life without the deceased person	Ongoing feelings of frustration, anxiety, depression, or despair that tinnitus will never go away
	Interference with daily functioning and relationships.	Difficulty habituating to the sound, with tinnitus dominating attention and mental space.
	May indicate complications such as Prolonged Grief Disorder (PGD).	May indicate co-occurring conditions such as anxiety disorders or depression.
**Delayed grief:**	Initial avoidance or suppression of grief.	Initial minimization, suppression, or unawareness of the emotional impact of tinnitus
	Emotional pain emerging months or even years after the loss.	Distress emerging later, often triggered by life stressors, increased tinnitus loudness, or reduced coping resources

## Data Availability

Data are available from W.S. on reasonable request.

## References

[1] De Ridder D., Schlee W., Vanneste S., Londero A., Weisz N., Kleinjung T., Shekhawat G.S., Elgoyhen A.B., Song J.J., Andersson G. (2021). Tinnitus and tinnitus disorder: Theoretical and operational definitions (an international multidisciplinary proposal). Prog. Brain Res..

[2] Tonndorf J. (1987). The analogy between tinnitus and pain: A suggestion for a physiological basis of chronic tinnitus. Hear. Res..

[3] Moller A.R. (1997). Similarities between chronic pain and tinnitus. Am. J. Otol..

[4] De Ridder D., Adhia D., Vanneste S. (2024). The brain’s duck test in phantom percepts: Multisensory congruence in neuropathic pain and tinnitus. Brain Res..

[5] De Ridder D., Elgoyhen A.B., Romo R., Langguth B. (2011). Phantom percepts: Tinnitus and pain as persisting aversive memory networks. Proc. Natl. Acad. Sci. USA.

[6] De Ridder D., Friston K., Sedley W., Vanneste S. (2023). A parahippocampal-sensory Bayesian vicious circle generates pain or tinnitus: A source-localized EEG study. Brain Commun..

[7] De Ridder D., Van de Heyning P. (2007). The Darwinian plasticity hypothesis for tinnitus and pain. Prog. Brain Res..

[8] De Ridder D., Vanneste S. (2021). The Bayesian brain in imbalance: Medial, lateral and descending pathways in tinnitus and pain: A perspective. Prog. Brain Res..

[9] De Ridder D., Adhia D., Vanneste S. (2021). The anatomy of pain and suffering in the brain and its clinical implications. Neurosci. Biobehav. Rev..

[10] Scholz J., Finnerup N.B., Attal N., Aziz Q., Baron R., Bennett M.I., Benoliel R., Cohen M., Cruccu G., Davis K.D. (2019). The IASP classification of chronic pain for ICD-11: Chronic neuropathic pain. Pain.

[11] Treede R.D., Rief W., Barke A., Aziz Q., Bennett M.I., Benoliel R., Cohen M., Evers S., Finnerup N.B., First M.B. (2019). Chronic pain as a symptom or a disease: The IASP Classification of Chronic Pain for the International Classification of Diseases (ICD-11). Pain.

[12] Cederroth C.R., Gallus S., Hall D.A., Kleinjung T., Langguth B., Maruotti A., Meyer M., Norena A., Probst T., Pryss R. (2019). Editorial: Towards an Understanding of Tinnitus Heterogeneity. Front. Aging Neurosci..

[13] Dawes P., Newall J., Stockdale D., Baguley D.M. (2020). Natural history of tinnitus in adults: A cross-sectional and longitudinal analysis. BMJ Open.

[14] Jarach C.M., Lugo A., Scala M., van den Brandt P.A., Cederroth C.R., Odone A., Garavello W., Schlee W., Langguth B., Gallus S. (2022). Global Prevalence and Incidence of Tinnitus: A Systematic Review and Meta-analysis. JAMA Neurol..

[15] Axelsson A., Ringdahl A. (1989). Tinnitus—A study of its prevalence and characteristics. Br. J. Audiol..

[16] Phoon W.H., Lee H.S., Chia S.E. (1993). Tinnitus in noise-exposed workers. Occup. Med..

[17] Swan A.A., Nelson J.T., Swiger B., Jaramillo C.A., Eapen B.C., Packer M., Pugh M.J. (2017). Prevalence of hearing loss and tinnitus in Iraq and Afghanistan Veterans: A Chronic Effects of Neurotrauma Consortium study. Hear. Res..

[18] Kreuzer P.M., Landgrebe M., Schecklmann M., Staudinger S., Langguth B., The TRI Database Study Group (2012). Trauma-associated tinnitus: Audiological, demographic and clinical characteristics. PLoS ONE.

[19] Kreuzer P.M., Landgrebe M., Vielsmeier V., Kleinjung T., De Ridder D., Langguth B. (2014). Trauma-associated tinnitus. J. head trauma Rehabil..

[20] Fagelson M. (2022). Tinnitus and Traumatic Memory. Brain Sci..

[21] Fagelson M.A. (2007). The association between tinnitus and posttraumatic stress disorder. Am. J. Audiol..

[22] Prewitt A., Harker G., Gilbert T.A., Hooker E., O’Neil M.E., Reavis K.M., Henry J.A., Carlson K.F. (2021). Mental Health Symptoms Among Veteran VA Users by Tinnitus Severity:A Population-based Survey. Mil. Med..

[23] Terhaag S., Phelps A., Howard A., O’Donnell M., Cowlishaw S. (2021). A longitudinal exploration of self-reported hearing loss, tinnitus and PTSD treatment outcomes in Australian veterans. Psychosom. Med..

[24] Bhatt J.M., Bhattacharyya N., Lin H.W. (2017). Relationships between tinnitus and the prevalence of anxiety and depression. Laryngoscope.

[25] Zhang H., Ji L., Wang L., Yin Z., Cen J., Guo Y. (2023). Clinical characteristics and psychoacoustic analysis of acute and chronic subjective tinnitus. Laryngoscope Investig. Otolaryngol..

[26] Vielsmeier V., Santiago Stiel R., Kwok P., Langguth B., Schecklmann M. (2020). From Acute to Chronic Tinnitus: Pilot Data on Predictors and Progression. Front. Neurol..

[27] Simoes J.P., Neff P.K.A., Langguth B., Schlee W., Schecklmann M. (2021). The progression of chronic tinnitus over the years. Sci. Rep..

[28] Vanneste S., van de Heyning P., De Ridder D. (2011). The neural network of phantom sound changes over time: A comparison between recent-onset and chronic tinnitus patients. Eur. J. Neurosci..

[29] Bonanno G.A. (2004). Loss, trauma, and human resilience: Have we underestimated the human capacity to thrive after extremely aversive events?. Am. Psychol..

[30] Wen F.H., Prigerson H.G., Chou W.C., Huang C.C., Hu T.H., Chiang M.C., Chuang L.P., Tang S.T. (2023). Comorbid Prolonged Grief, PTSD, and Depression Trajectories for Bereaved Family Surrogates. JAMA Netw. Open.

[31] Langguth B., Goodey R., Azevedo A., Bjorne A., Cacace A., Crocetti A., Del Bo L., De Ridder D., Diges I., Elbert T. (2007). Consensus for tinnitus patient assessment and treatment outcome measurement: Tinnitus Research Initiative meeting. Prog. Brain Res..

[32] Hiller W., Goebel G. (2004). Rapid Assessment of tinnitus-related psychological distress using the Mini-TQ. Int J Audiol..

[33] Mohan A., Leong S.L., De Ridder D., Vanneste S. (2022). Symptom dimensions to address heterogeneity in tinnitus. Neurosci. Biobehav. Rev..

[34] De Ridder D., Moller A., Langguth B., De Ridder D., Kleinjung T. (2011). A heuristic pathophysiological model of tinnitus. Chapter 21. Textbook of Tinnitus.

[35] Masten A.S., Barnes A.J. (2018). Resilience in Children: Developmental Perspectives. Children.

[36] Utzon-Frank N., Breinegaard N., Bertelsen M., Borritz M., Eller N.H., Nordentoft M., Olesen K., Rod N.H., Rugulies R., Bonde J.P. (2014). Occurrence of delayed-onset post-traumatic stress disorder: A systematic review and meta-analysis of prospective studies. Scand. J. Work. Environ. Health.

[37] Xin F., Li Q., Guan F., Suo M., Yang J., Li D., Zhao C. (2022). The study on psychological resilience of tinnitus and associated influencing factors. J. Otol..

[38] Martins M.L., Galdino M.K.C., Fernandez-Calvo B., Branco-Barreiro F.C.A., Fernandes T.P., da Rosa M.R.D. (2022). Perception of Tinnitus: Direct and Indirect Effects of Resilience, Personality Traits, and Psychiatric Symptoms. J. Am. Acad. Audiol..

[39] Langguth B., Kleinjung T., Fischer B., Hajak G., Eichhammer P., Sand P.G. (2007). Tinnitus severity, depression, and the big five personality traits. Prog. Brain Res..

[40] Wallhausser-Franke E., Delb W., Balkenhol T., Hiller W., Hormann K. (2014). Tinnitus-related distress and the personality characteristic resilience. Neural Plast..

[41] Chen L., Liu A. (2015). The Incidence of Posttraumatic Stress Disorder After Floods: A Meta-Analysis. Disaster Med. Public Health Prep..

[42] Lim I., Tam W.W.S., Chudzicka-Czupala A., McIntyre R.S., Teopiz K.M., Ho R.C., Ho C.S.H. (2022). Prevalence of depression, anxiety and post-traumatic stress in war- and conflict-afflicted areas: A meta-analysis. Front. Psychiatry.

[43] Yuan K., Gong Y.M., Liu L., Sun Y.K., Tian S.S., Wang Y.J., Zhong Y., Zhang A.Y., Su S.Z., Liu X.X. (2021). Prevalence of posttraumatic stress disorder after infectious disease pandemics in the twenty-first century, including COVID-19: A meta-analysis and systematic review. Mol. Psychiatry.

[44] Yuan M.D., Wang Z.Q., Fei L., Zhong B.L. (2022). Prevalence of prolonged grief disorder and its symptoms in Chinese parents who lost their only child: A systematic review and meta-analysis. Front. Public Health.

[45] Lundorff M., Holmgren H., Zachariae R., Farver-Vestergaard I., O’Connor M. (2017). Prevalence of prolonged grief disorder in adult bereavement: A systematic review and meta-analysis. J. Affect. Disord..

[46] Zareiyan A., Sahebi A., Nejati-Zarnaqi B., Mosaed R., Ozouni-Davaji R.B. (2024). The prevalence of prolonged grief disorder (PGD) after the natural disasters: A systematic review and meta-analysis. Public Health Pract..

[47] Yuan M.D., Liu J.F., Zhong B.L. (2024). Prevalence of prolonged grief disorder and its symptoms among bereaved individuals in China: A systematic review and meta-analysis. Gen. Psychiatr..

[48] Bonanno G.A., Westphal M., Mancini A.D. (2011). Resilience to loss and potential trauma. Annu. Rev. Clin. Psychol..

[49] Kristensen P., Weisaeth L., Heir T. (2012). Bereavement and mental health after sudden and violent losses: A review. Psychiatry.

[50] Sanchez T.G., Valim C.C.A., Schlee W. (2021). Long-lasting total remission of tinnitus: A systematic collection of cases. Prog Brain Res..

[51] Morstead T., Rights J.D., Sin N.L., DeLongis A. (2024). Predictors of Complicated Grief During the COVID-19 Pandemic: A Cross-Classified Analysis. Omega (Westport).

[52] Goetter E., Bui E., Horenstein A., Baker A.W., Hoeppner S., Charney M., Simon N.M. (2019). Five-factor model in bereaved adults with and without complicated grief. Death Stud..

[53] de Lang T.A., Buyukcan-Tetik A., de Jong P.J., Lancel M., Eisma M.C. (2024). Trajectories of insomnia following bereavement. Sleep Med..

[54] Garrouste-Orgeas M., Marche V., Pujol N., Michel D., Evin A., Fossez-Diaz V., Perruchio S., Vanbesien A., Verlaine C., Copel L. (2023). Incidence and risk factors of prolonged grief in relatives of patients with terminal cancer in French palliative care units: The Fami-Life multicenter cohort study. Palliat. Support. Care.

[55] Basso L., Boecking B., Brueggemann P., Pedersen N.L., Canlon B., Cederroth C.R., Mazurek B. (2020). Gender-Specific Risk Factors and Comorbidities of Bothersome Tinnitus. Front. Neurosci..

[56] Bonanno G.A., Kaltman S. (2001). The varieties of grief experience. Clin. Psychol. Rev..

[57] Rosenhall U., Karlsson A.K. (1991). Tinnitus in old age. Scand. Audiol..

[58] Rosenhall U. (2003). The influence of ageing on noise-induced hearing loss. Noise Health.

[59] Turner J.G., Larsen D. (2016). Effects of noise exposure on development of tinnitus and hyperacusis: Prevalence rates 12 months after exposure in middle-aged rats. Hear. Res..

[60] Ridder D.D., Schlee W., Langguth B., De Ridder D., Vanneste S., Kleinjung T., Moller A. (2024). Trauma-associated Tinnitus. Chapter 38. Textbook of Tinnitus.

[61] Patil J.D., Alrashid M.A., Eltabbakh A., Fredericks S. (2023). The association between stress, emotional states, and tinnitus: A mini-review. Front. Aging Neurosci..

[62] De Ridder D., Vanneste S., Congedo M. (2011). The distressed brain: A group blind source separation analysis on tinnitus. PLoS ONE.

[63] De Ridder D., Vanneste S., Song J.J., Adhia D. (2022). Tinnitus and the triple network model: A perspective. Clin. Exp. Otorhinolaryngol..

[64] Peters A., McEwen B.S., Friston K. (2017). Uncertainty and stress: Why it causes diseases and how it is mastered by the brain. Prog. Neurobiol..

[65] Seeley W.W., Menon V., Schatzberg A.F., Keller J., Glover G.H., Kenna H., Reiss A.L., Greicius M.D. (2007). Dissociable intrinsic connectivity networks for salience processing and executive control. J. Neurosci..

[66] Glaser R., Kiecolt-Glaser J.K. (2005). Stress-induced immune dysfunction: Implications for health. Nat. Rev. Immunol..

[67] Kim J., Yoon S., Lee S., Hong H., Ha E., Joo Y., Lee E.H., Lyoo I.K. (2020). A double-hit of stress and low-grade inflammation on functional brain network mediates posttraumatic stress symptoms. Nat. Commun..

[68] Ciminelli P., Machado S., Palmeira M., Carta M.G., Beirith S.C., Nigri M.L., Mezzasalma M.A., Nardi A.E. (2018). Tinnitus: The Sound of Stress?. Clin. Pract. Epidemiol. Ment. Health.

[69] Elarbed A., Fackrell K., Baguley D.M., Hoare D.J. (2021). Tinnitus and stress in adults: A scoping review. Int. J. Audiol..

[70] Straub R.H. (2017). The brain and immune system prompt energy shortage in chronic inflammation and ageing. Nat. Rev. Rheumatol..

[71] Turunen-Taheri S., Carlsson P.I., Ternevall E., Hellstrom S. (2023). Mental Fatigue in Patients with Hearing Loss and/or Tinnitus Undergoing Audiological Rehabilitation—A Pilot Study. J. Clin. Med..

[72] Norena A.J. (2011). An integrative model of tinnitus based on a central gain controlling neural sensitivity. Neurosci. Biobehav. Rev..

[73] Wang W., Zhang L.S., Zinsmaier A.K., Patterson G., Leptich E.J., Shoemaker S.L., Yatskievych T.A., Gibboni R., Pace E., Luo H. (2019). Neuroinflammation mediates noise-induced synaptic imbalance and tinnitus in rodent models. PLoS Biol..

[74] De Ridder D., Vanneste S., Weisz N., Londero A., Schlee W., Elgoyhen A.B., Langguth B. (2014). An integrative model of auditory phantom perception: Tinnitus as a unified percept of interacting separable subnetworks. Neurosci. Biobehav. Rev..

[75] Sedley W., Friston K.J., Gander P.E., Kumar S., Griffiths T.D. (2016). An Integrative Tinnitus Model Based on Sensory Precision. Trends Neurosci..

[76] De Ridder D., Vanneste S., Freeman W. (2014). The Bayesian brain: Phantom percepts resolve sensory uncertainty. Neurosci. Biobehav. Rev..

[77] Weisz N., Hartmann T., Dohrmann K., Schlee W., Norena A. (2006). High-frequency tinnitus without hearing loss does not mean absence of deafferentation. Hear. Res..

[78] Lee S.Y., Chang M., Kwon B., Choi B.Y., Koo J.W., Moon T., De Ridder D., Vanneste S., Song J.J. (2021). Is the posterior cingulate cortex an on-off switch for tinnitus?: A comparison between hearing loss subjects with and without tinnitus. Hear. Res..

[79] De Ridder D., Vanneste S. (2024). Thalamocortical dysrhythmia and reward deficiency syndrome as uncertainty disorders. Neuroscience.

[80] Ryan M., Ryznar R. (2022). The Molecular Basis of Resilience: A Narrative Review. Front. Psychiatry.

[81] Hoffmann A., Sportelli V., Ziller M., Spengler D. (2017). Epigenomics of Major Depressive Disorders and Schizophrenia: Early Life Decides. Int. J. Mol. Sci..

[82] Raabe F.J., Spengler D. (2013). Epigenetic Risk Factors in PTSD and Depression. Front. Psychiatry.

[83] Vanneste S., Alsalman O., De Ridder D. (2018). COMT and the neurogenetic architecture of hearing loss induced tinnitus. Hear. Res..

[84] Vanneste S., Mohan A., De Ridder D., To W.T. (2021). The BDNF Val^66^Met polymorphism regulates vulnerability to chronic stress and phantom perception. Prog. Brain Res..

[85] Clifford R.E., Maihofer A.X., Chatzinakos C., Coleman J.R.I., Daskalakis N.P., Gasperi M., Hogan K., Mikita E.A., Stein M.B., Tcheandjieu C. (2024). Genetic architecture distinguishes tinnitus from hearing loss. Nat. Commun..

[86] Hong Y., Wang Y., Shu W. (2025). Deciphering the genetic underpinnings of neuroticism: A Mendelian randomization study of druggable gene targets. J. Affect. Disord..

[87] Nagel M., Jansen P.R., Stringer S., Watanabe K., de Leeuw C.A., Bryois J., Savage J.E., Hammerschlag A.R., Skene N.G., Munoz-Manchado A.B. (2018). Meta-analysis of genome-wide association studies for neuroticism in 449,484 individuals identifies novel genetic loci and pathways. Nat. Genet..

[88] Austin-Zimmerman I., Levey D.F., Giannakopoulou O., Deak J.D., Galimberti M., Adhikari K., Zhou H., Denaxas S., Irizar H., Kuchenbaecker K. (2023). Genome-wide association studies and cross-population meta-analyses investigating short and long sleep duration. Nat. Commun..

[89] Watanabe K., Jansen P.R., Savage J.E., Nandakumar P., Wang X., Hinds D.A., Gelernter J., Levey D.F., Polimanti R., 23andMe Research Team (2022). Genome-wide meta-analysis of insomnia prioritizes genes associated with metabolic and psychiatric pathways. Nat. Genet..

[90] Conrad I., Kleinstauber M., Jasper K., Hiller W., Andersson G., Weise C. (2015). The Role of Dysfunctional Cognitions in Patients With Chronic Tinnitus. Ear Hear..

[91] Simoes J., Schlee W., Schecklmann M., Langguth B., Farahmand D., Neff P. (2019). Big Five Personality Traits are Associated with Tinnitus Improvement Over Time. Sci. Rep..

[92] Herrera-Rivero M., Garvert L., Horn K., Lobner M., Weitzel E.C., Stoll M., Lichtner P., Teismann H., Teumer A., Van der Auwera S. (2025). A meta-analysis of genome-wide studies of resilience in the German population. Mol. Psychiatry.

[93] Coelho D.R.A., Renet C., Lopez-Rodriguez S., Cassano P., Vieira W.F. (2024). Transcranial photobiomodulation for neurodevelopmental disorders: A narrative review. Photochem. Photobiol. Sci..

[94] Zielinski M.R., Systrom D.M., Rose N.R. (2019). Fatigue, Sleep, and Autoimmune and Related Disorders. Front. Immunol..

[95] Pape K., Tamouza R., Leboyer M., Zipp F. (2019). Immunoneuropsychiatry-novel perspectives on brain disorders. Nat. Rev. Neurol..

[96] Basso L., Boecking B., Neff P., Brueggemann P., El-Ahmad L., Brasanac J., Rose M., Gold S.M., Mazurek B. (2022). Negative Associations of Stress and Anxiety Levels With Cytotoxic and Regulatory Natural Killer Cell Frequency in Chronic Tinnitus. Front. Psychol..

[97] Mennink L.M., Aalbers M.W., van Dijk P., van Dijk J.M.C. (2022). The Role of Inflammation in Tinnitus: A Systematic Review and Meta-Analysis. J. Clin. Med..

[98] Passos I.C., Vasconcelos-Moreno M.P., Costa L.G., Kunz M., Brietzke E., Quevedo J., Salum G., Magalhaes P.V., Kapczinski F., Kauer-Sant’Anna M. (2015). Inflammatory markers in post-traumatic stress disorder: A systematic review, meta-analysis, and meta-regression. Lancet Psychiatry.

[99] Peruzzolo T.L., Pinto J.V., Roza T.H., Shintani A.O., Anzolin A.P., Gnielka V., Kohmann A.M., Marin A.S., Lorenzon V.R., Brunoni A.R. (2022). Inflammatory and oxidative stress markers in post-traumatic stress disorder: A systematic review and meta-analysis. Mol. Psychiatry.

[100] Prasad A., Chaichi A., Kelley D.P., Francis J., Gartia M.R. (2019). Current and future functional imaging techniques for post-traumatic stress disorder. RSC Adv..

[101] Seiler A., von Kanel R., Slavich G.M. (2020). The Psychobiology of Bereavement and Health: A Conceptual Review From the Perspective of Social Signal Transduction Theory of Depression. Front. Psychiatry.

[102] Schultze-Florey C.R., Martinez-Maza O., Magpantay L., Breen E.C., Irwin M.R., Gundel H., O’Connor M.F. (2012). When grief makes you sick: Bereavement induced systemic inflammation is a question of genotype. Brain Behav. Immun..

[103] O’Connor M.F., Schultze-Florey C.R., Irwin M.R., Arevalo J.M.G., Cole S.W. (2014). Divergent gene expression responses to complicated grief and non-complicated grief. Brain Behav. Immun..

[104] Martin-Lagos J., Bernal-Robledano A., Perez-Carpena P., Lamolda M., Escalera-Balsera A., Frejo L., Lopez-Escamez J.A. (2024). Phenotypic spectrum of tinnitus patients bearing rare *ANK2* gene variants. Eur. Arch. Otorhinolaryngol..

[105] Perez-Carpena P., Lopez-Escamez J.A., Gallego-Martinez A. (2024). A Systematic Review on the Genetic Contribution to Tinnitus. J. Assoc. Res. Otolaryngol..

[106] Howe A.S., Buttenschon H.N., Bani-Fatemi A., Maron E., Otowa T., Erhardt A., Binder E.B., Gregersen N.O., Mors O., Woldbye D.P. (2016). Candidate genes in panic disorder: Meta-analyses of 23 common variants in major anxiogenic pathways. Mol. Psychiatry.

[107] Naik R.R., Sotnikov S.V., Diepold R.P., Iurato S., Markt P.O., Bultmann A., Brehm N., Mattheus T., Lutz B., Erhardt A. (2018). Polymorphism in Tmem132d regulates expression and anxiety-related behavior through binding of RNA polymerase II complex. Transl. Psychiatry.

[108] Vanneste S., Figueiredo R., De Ridder D. (2012). Treatment of tinnitus with cyclobenzaprine: An open-label study. Int. J. Clin. Pharmacol. Ther..

[109] Hilton M.P., Zimmermann E.F., Hunt W.T. (2013). Ginkgo biloba for tinnitus. Cochrane Database Syst. Rev..

